# Laparoscopic Excision of Patent Processus Vaginalis for Pediatric Hydroceles

**DOI:** 10.7759/cureus.18416

**Published:** 2021-10-01

**Authors:** Behrouz Banieghbal

**Affiliations:** 1 Division of Paediatric Surgery, Stellenbosch University, Cape Town, ZAF

**Keywords:** hydrocele, multi-modality pain management, scar less, surgical techniques, inguinal hernia repair, advanced laparoscopy

## Abstract

Background

The standard surgical practice for pediatric hydrocele is resection and ligation of the patent processus vaginalis (PPV). Non-ligation of PPV for pediatric hydrocele is another possibility that can be repaired laparoscopically.

Material & methods

A retrospective study was undertaken over 10 years (Jan 2011-Feb 2020), of a case series of boys with hydroceles that underwent laparoscopic PPV (Lap PPV) excision. Exclusion criteria were for parents who requested open surgery (10 cases) or an omental plug noted at the PPV site during laparoscopy (one case). Laparoscopic PPV excision was performed via a transperitoneal approach.

Results

There were 43 cases of Lap PP excision, including three recurrences after open surgery. There were no conversions, complications, or recurrences in any patients. The average operative time for unilateral cases was 21 mins (range 15-30 mins). Three concurrent contra-lateral hydroceles were noted and resected during the primary procedure. Time to regular activity was within one day. There was no visible scar or recurrence after Lap PPV at six months post-surgery review.

Conclusion

Lap PPV excision appears to be at least equivalent to the “open and ligation” approach. During laparoscopy, both internal rings are assessed for a PPV.

By avoiding an inguinal incision(s), a better cosmetic result is possible. It is conceivably safer than open surgery in recurrent cases.

## Introduction

A patent processus vaginalis (PPV) is the leading cause of indirect inguinal hernia and hydrocele in the pediatric population. Hydrocele is a common congenital anomaly reported in 60% of newborn males [1].

Traditionally, hydrocele surgery can be deferred until two years of age because a spontaneous closure of PPV often occurs [2]. The standard of surgical management for pediatric hydrocele has been an open groin approach, with resection of the PPV with proximal ligation of the hernia sac.

Excision of the PPV without ligation of the sac can be done in orchidopexy for undescended testis or during inguinal herniotomy. It is proposed in this presentation that non-ligation of the PPV for the management of hydroceles is possible. It will simplify it, thus rendering the procedure to a laparoscopic excision of PPV (Lap PPV).

A case series of children is presented who underwent laparoscopic excision of PPV (Lap PPV).

## Materials and methods

Forty-three consecutive children underwent surgery for primary hydrocele or recurrent hydroceles (Jan 2011 and Feb 2020) with Laparoscopic PPV (Lap PPV) excision. The peri-operative data were collected and then analyzed from a constructed hernia database.

The index surgical procedures were carried out by, one surgeon (BB), in two teachings and three private hospitals in South Africa.

In the consent process, parents had been informed that the open and laparoscopic procedures were fundamentally the same, and the main difference was a reduction in the size of the scar, with a possible lessening of pain post-operatively.

The inclusion criteria were all prepubescent boys, 2 to 12 years old, with unilateral or bilateral hydroceles. There were no exclusion criteria unless parents preferred an open procedure for personal reasons or an omental plug noted during laparoscopy at the internal ring, which required a laparoscopic inguinal herniotomy.

For the procedure, the steps followed are like laparoscopic inguinal herniotomy. The last step of suturing of the ring is excluded. A short description follows:

Lap PPV excision was done via trans-peritoneal approach with insertion of a 5-mm trocar in the umbilical area by open Hasson technique. Pneumoperitoneum was attained with CO_2_ insufflation set at 8-10 mmHg. A 5-mm 30° scope was used to visualize and confirm the presence of PPV. If insufflation caused scrotal swelling, with visual confirmation of PPV on both sides, the procedure was done as a bilateral case.

**Figure 1:**
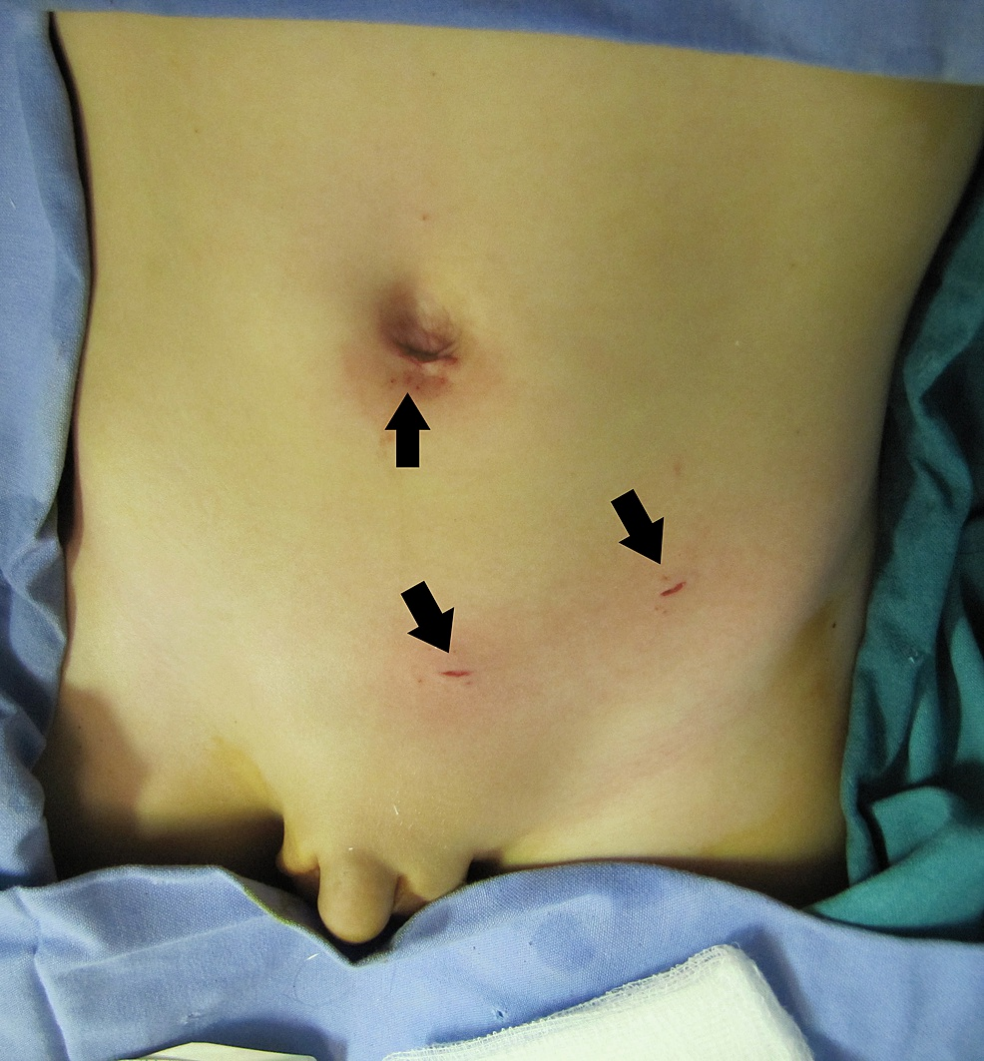
Entry points for the umbilical port and 3mm instruments (solid black arrows) for an 8-year-old boy with left hydrocele. Entry points for the umbilical port and 3mm instruments (solid black arrows)

Two 3-mm instruments were placed, percutaneously, about 10 cm away from the internal ring in a triangular method commonly performed for ergonomic convenience like all laparoscopic procedures (Figure 1).

For a unilateral PPV case, two 3-mm instruments were inserted, a curved grasper near the midline and curved scissors on the latter aspect of the abdominal cavity. In bilateral cases, after completion of one side, an additional 3-mm incision was made on the other side and lateral to the internal ring; the mid-line trocar often reached the other side.

**Figure 2:**
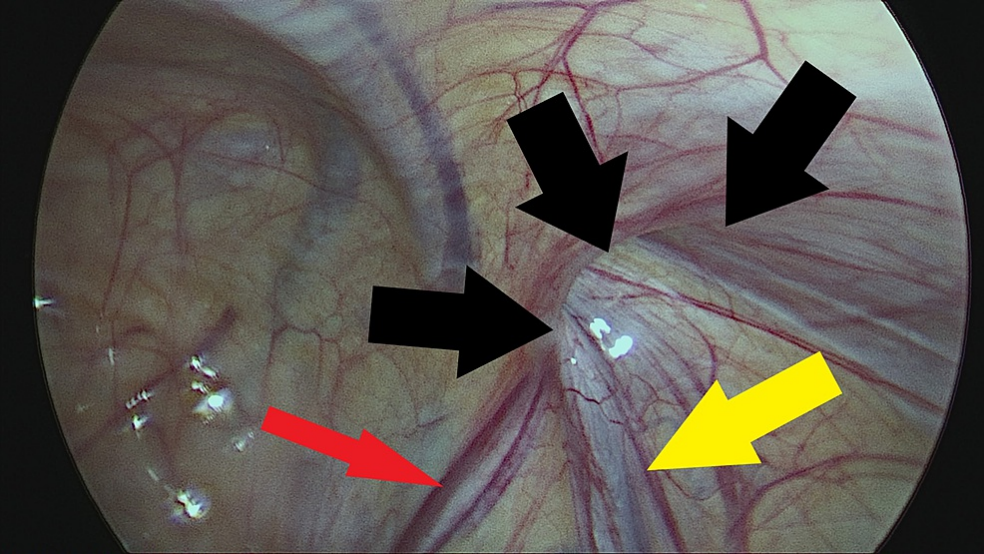
Confirmation of patent processus vaginalis (solid black arrows), vas deferens (solid red arrow) and spermatic vessels (solid yellow arrow). Inspection of the right internal ring by laparoscopy.

**Figure 3:**
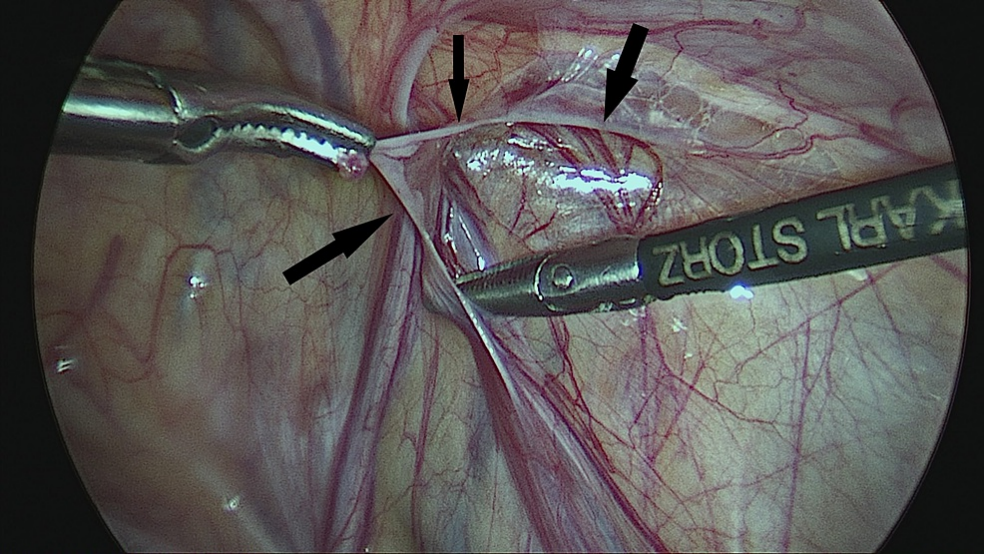
Sharp dissection of patent processus on its lateral aspect by a 3-mm scissors (solid black arrows). Steps in surgical excision

**Figure 4:**
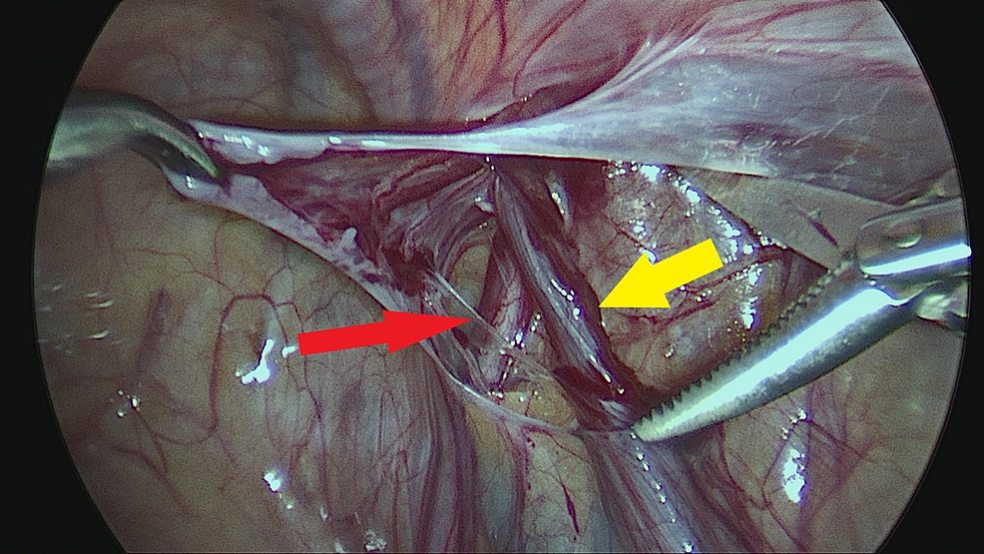
Further sharp/blunt dissection and separation of vas deferens (solid red arrow) and testicular vessels (solid yellow arrow). Steps in surgical excision

**Figure 5:**
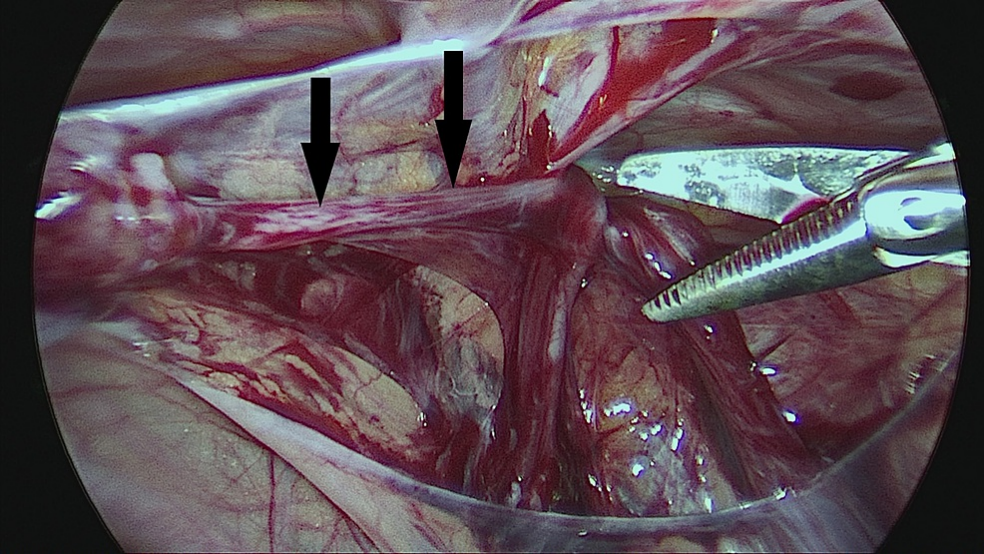
Gentle blunt retraction of patent processus (solid black arrows) prior to sharp excision. Steps in surgical excision

**Figure 6:**
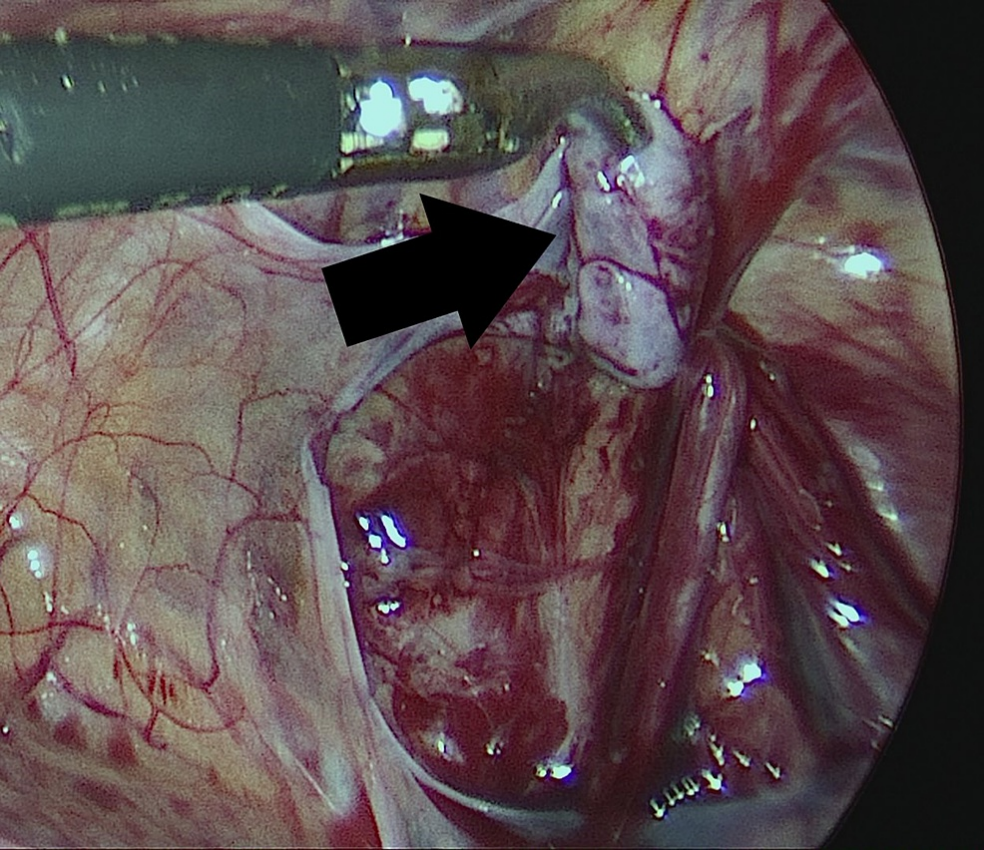
Excision of a short segment of patent processus (solid black arrow). The final step in surgical excision

The PPV was sharply incised on its lateral aspect and then all around by further blunt dissection. A short segment of the PPV, about 2 cm long, was mobilized away from vas and vessels and cut sharply with scissors without ligation (Figures 2-6).

Pneumoperitoneum was deflated, and wounds closed in the standard way; 1% bupivacaine local anesthetic was injected as a local block in all the incisions.

The patients were treated as day-case surgery. Oral ibuprofen/paracetamol (acetaminophen) combination was recommended 8-hourly for post-operative analgesia. Parents were advised to give three divided doses of oral analgesia daily and continue if the child was in pain for up to three days.

All patients were followed up at one and six months after surgery. Those who could not attend in person were contacted telephonically.

## Results

Lap PPV was used to correct the hydrocele in 40 boys, including three bilateral cases. Additionally, three recurrent cases were referred, after open surgery by other surgeons, and managed laparoscopically. There were no conversions, no significant complications, and no recurrences in this cohort with a six-month follow-up (Table 1).

**Table 1:** Summary of the cases and results Simplified table with outcomes * Student Test was not significant (i.e., p-value > 0.05) PPV: Patent processus vaginalis

	Cases	Age in years (average)	Average surgical time (mins)	Morbidity at 1 and 6 months	Average oral analgesic doses/patient	Time to full activity (hours)
Lap PPV (unilateral)	37	2.9	22	0	4.3	24
Lap PPV (Bilateral)	3	2.5	30	0	4.5	25
Lap PPV (after previous open surgery)	3	3	22	0	4.3	24
Omental Plug at PPV	1	2.2	30	0	4.3	24
Open PPV	10	3	20	0	4.8	26
Probability (p) value		0.8*	0.7* (excluding bilateral cases and omental plug)	0.9*	0.7*	0.6*

The average age of patients was 2.9 years (range 2-6 years). One known bilateral case was repaired and two contra-lateral PPV were identified on laparoscopy and managed accordingly.

Lap PPV operative time was about 22 mins long (range 15-25 mins), excluding three bilateral cases, which took an extra 15 minutes.

The children required an average of 4.3 analgesic doses, which consisted of 8-hourly doses of ibuprofen and paracetamol mixture (range 2-6 doses). Eight older children, who could verbalize, pointed to their umbilical wound as the cause of their post-operative pain. After changing the insertion of the umbilical port from horizontal to vertical incision, there was no further report of discomfort in that area in a further two older boys.

Excluded patients were 10 parents who requested open surgery for personal reasons. In the Lap PPV group, one patient had an omental plug that was obstructing the internal ring; laparoscopic excision of PPV and suture ligation of the remnant was carried out in that specific case.

**Figure 7:**
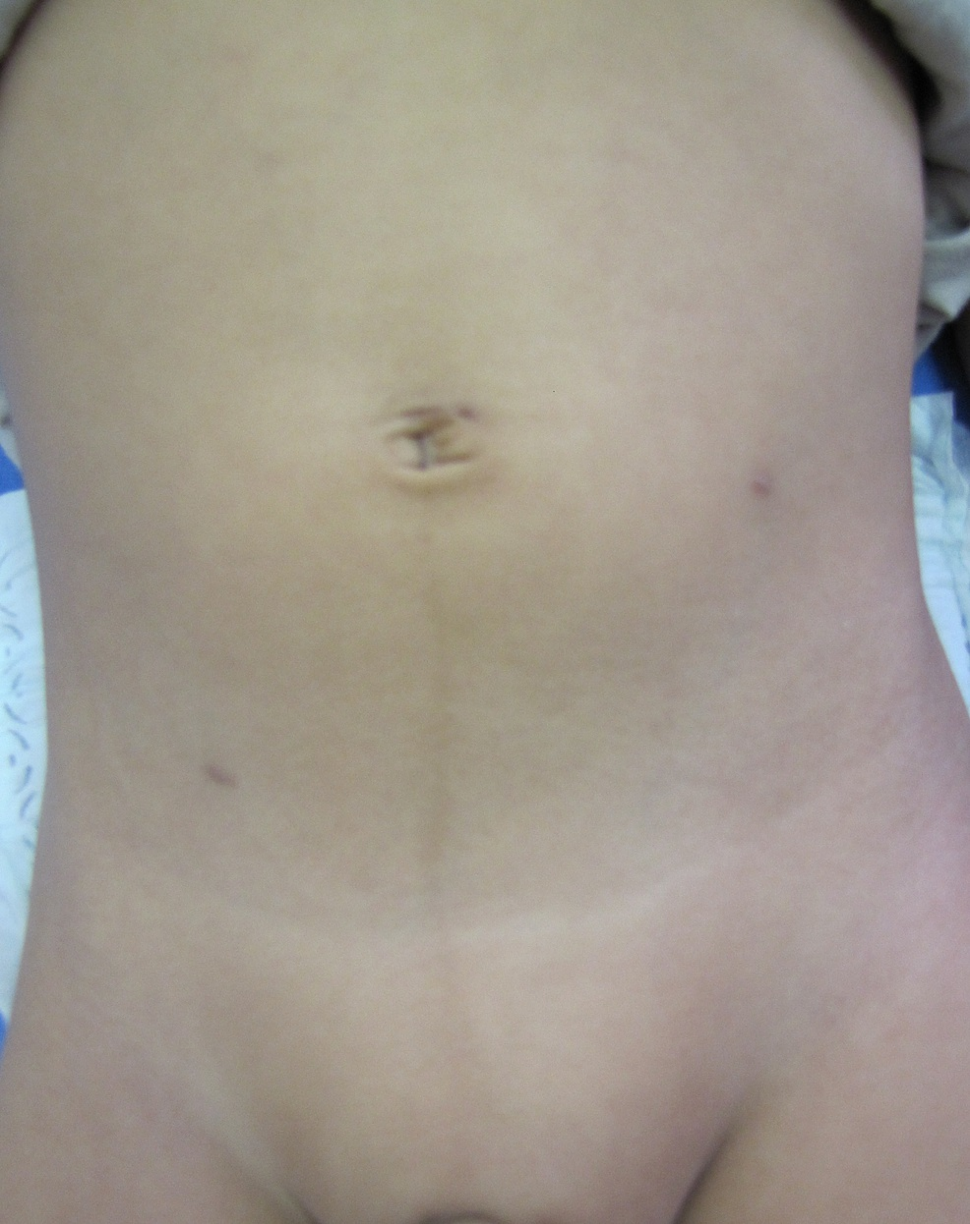
Post-operative view of the abdomen, one-week post-surgery for a 2-year-old boy with left-sided hydrocele. One-week post-operative view of the abdomen in a 2-year-old boy.

**Figure 8:**
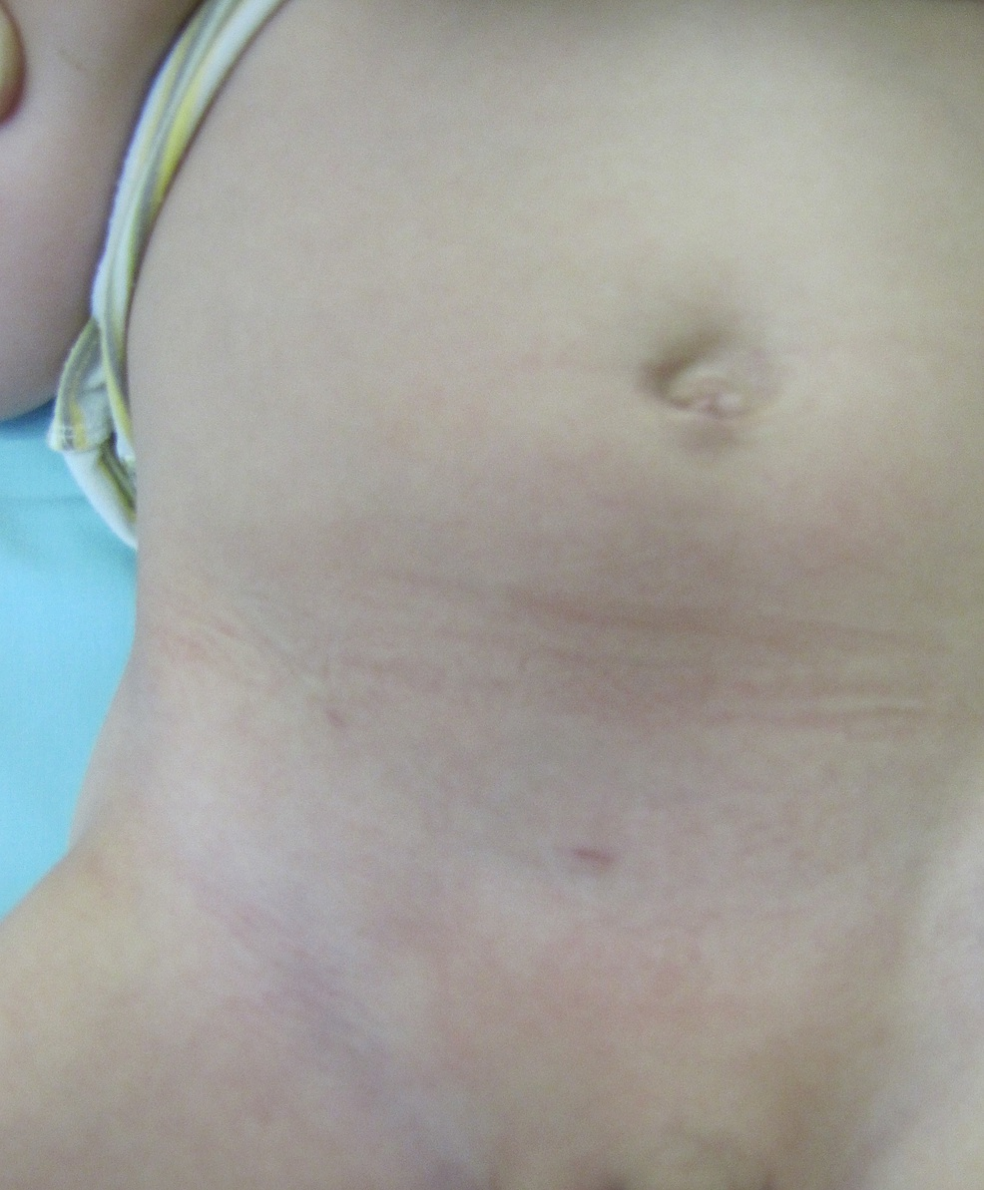
Post-surgery view of the abdomen at 6 months in a 5-year-old with right-sided hydrocele. Scars are barely visible in a different patient at six months post-surgery.

There was minimal scar with Lap PPV at one-week post-surgery (Figure 7) and six months later, minimal scars were visible (Figure 8).

**Figure 9:**
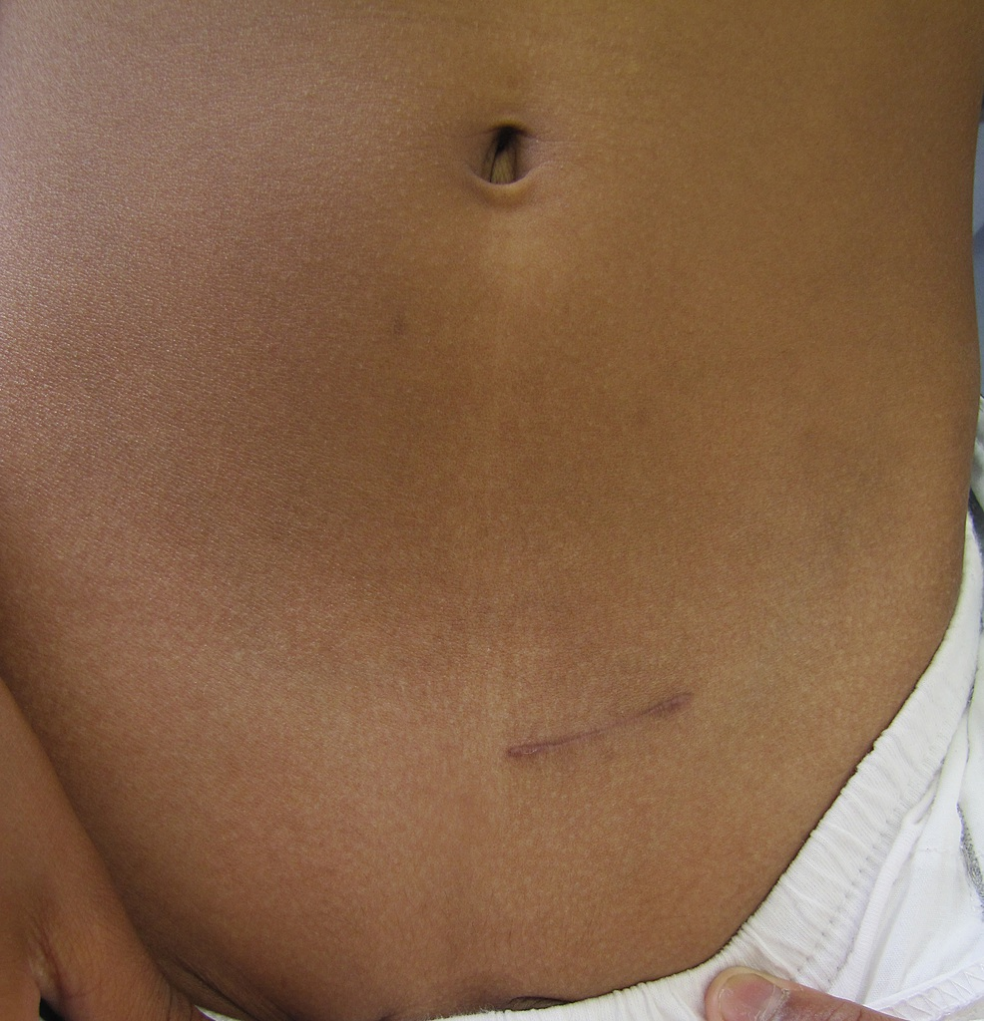
Left inguinal scar after open surgery, in a 3-year-old boy, six months post-surgery for a left hydrocele. Left inguinal scar after open surgery, six months later.

This was compared to patients who underwent open surgery and seen after six months (Figure 9).

According to parents, the time to regular activity, i.e., the expected degree of physical activity for each child after surgery, compared to before surgery, was about 24 hours in all patients.

There was no statistical difference noted between 43 laparoscopic cases versus 10 patients in the open group (Table 1).

## Discussion

The incidence of hydrocele in males at birth is high. However, it reduces to less than 0.5% by two years and does not decrease further with age [1,2].

The procedure can be delayed for many months or years if consent is not given. Currently, there is no published evidence that older boys or teenagers with non-treated hydrocele would develop complications such as infertility or Leydig's cell malfunction.

For decades, open surgical PPV resection and ligation at its proximal part has been the gold standard for definitive treatment of hydroceles [3]. This procedure is identical to herniotomy; the main difference is that PPV is small in caliber compared to inguinal hernia [4,5].

Therefore, excluding a section of PPV and non-ligation is possibly as adequate as ligation in inguinal herniotomy [6,7]. Laparoscopic excision follows the same concept except for its trans-peritoneal approach and unique instrumentations.

It is postulated that a likely reason for PPV non-closure is the presence of smooth muscle that may prevent or at least delay PPV's closure at birth, resulting in inguinal hernia [8]. However, in cases of hydrocele, the smooth muscle bundles are distributed as a patchy bundle, which explains its gradual closure by age [9].

The umbilical incision remained the primary source of pain in patients, despite the local injection of anesthetic agents. It is conceivable that a curved transverse sub-umbilical incision made in linea alba for placement of the first trocar allowed for minor movement in the mid-line fascia that can cause post-operative pain.

Later in the study, a vertical incision was made in linea alba to reduce tenderness around the umbilicus. This technique was less considered to be less painful in older boys, but the numbers were not enough for any meaningful analysis. A separate evaluation is underway to assess this minor change in the first trocar insertion in other routine laparoscopic procedures.

Operative simplicity and fine dissection are required to separate the cord vessels and vas deferens from the PPV; magnification obtained by a laparoscope is 6-8 times higher than the naked eye [10]. It is expected that an injury to vas deferens and vessels should occur less frequently, but a larger trial with less experienced trainees is needed to qualify that assertion.

This study has at least four limitations:

1) It is a relatively small cohort, and follow-up was concluded at six months; a longer follow-up as of two years would have been ideal.

2) A few parents opted for open surgery for other reasons. This could be considered a selection bias, even if it was the parents' decision and not the surgeon's.

3) Pain assessment and level of activity were done by the parents who may have been inclined toward laparoscopic surgery (information bias).

4) Confounding bias is probable because children may hesitate to inform their parents or doctor(s) regarding pain due to cultural reasons.

## Conclusions

Lap PPV excision and non-ligation appear to be at least equivalent to the open approach, but a multicentric large randomized control trial is needed to confirm the utility of this approach. The main advantage of laparoscopy for primary repair of hydrocele is for a better cosmetic outcome and inspection of the contralateral internal ring.
